# Genetic diversity, viraemic and aminotransferases levels in chronic infected hepatitis B patients from Cameroon

**DOI:** 10.1186/s13104-016-1916-7

**Published:** 2016-02-22

**Authors:** Kukwah Anthony Tufon, Henry Dilonga Meriki, Damian Nota Anong, Herbert Afegenwi Mbunkah, Theresa Nkuo-Akenji

**Affiliations:** Department of Microbiology and Parasitology, Faculty of Science, University of Buea, Buea, Cameroon; Microbiology Unit, Buea Regional Hospital, Buea, Southwest Region Cameroon; BioCollections Worldwide Inc., Miami, FL USA; Clinical Diagnostic Laboratory, Faculty of Science, University of Buea, P.O. Box 63, Buea, Cameroon; Internal Control and Evaluation, University of Buea, Buea, Cameroon

**Keywords:** HBV, AST, ALT, Viral load, HBV genotype

## Abstract

**Background:**

HBV infection annually accounts for 1 million deaths worldwide as a result of cirrhosis, liver failure, and hepatocellular carcinoma. In addition to varying responses to antiviral therapy, HBV genotypes have also been shown to be associated with different pattern of disease progression. Despite a high HBV prevalence of >8 %, very few studies have been carried out in Cameroon to determine the genotype distribution across the country. The aim of this study was to determine the prevalent genotypes, level of viraemia and correlate these parameters with liver enzymes known to be the most affordable and widely used biomarkers for monitoring disease progression in Cameroon.

**Methods:**

This was a hospital-community based study in which 81 participants who had been previously diagnosed of HBV were recruited and screened for HIV, HCV (for exclusion) and HBsAg for confirmation. Fifty known negative cases for HIV, HBV and HCV were tested and recruited to be used as healthy controls. Viral load and genotyping was performed only for HBV-mono infected cases using the Abbott Real*Time* HBV automated *m*2000 system and INNO-LiPA HBV Genotyping assay respectively. Liver enzymes were measured by spectrophotometry on both hepatitis B positive and healthy control cases.

**Results:**

The mean alanine aminotransferase (ALT) and aspartate aminotransferase (AST) levels were significantly higher (p < 0.001) in HBV infected patients than “healthy controls”. Of the 81 HBV infected cases viral load was detected in 76 (93.8 %) with mean viral load of 120,807 IU/ml ± 440,159 SD. Mean viral load was significantly different in patients with abnormal AST and ALT when compared with patients who had normal ALT and AST. The identified genotypes in order of prevalence were A (47.4 %), E (39.5 %), C/E (3.9 %) A/C (2.6 %), A/E (2.6 %), B (1.3 %), A/B (1.3 %) and B/C (1.3 %).

**Conclusion:**

Genotype E was significantly associated with higher mean viral load and mean AST levels. However, aminotransferase levels may not be a good marker for HBV disease progression as some patients could have normal levels but still present with very high viral loads and therefore, remain active HBV infection with possible high transmission.

## Background

Hepatitis B virus (HBV) which is a global health problem, presents serological evidence of infection in approximately one-third of the world’s population with over 350–400 million people being chronic HBV surface antigen (HBsAg) carriers [1]. Out of this number, 65 million reside in Africa and this implies that Africa, with 12 % of the world’s population, carries approximately 18 % of the global burden of HBV infection [2]. In addition to the difficulties encountered in controlling the spread of the disease, HBV still remains a health hazard partly because the virus exhibits different genotypes and sub genotypes in different geographic regions of the world [3].

Several viral factors like HBV genotype, viral load and specific viral mutations have been associated with disease progression ranging from acute to chronic infection which may later on cause liver cirrhosis and subsequent Hepatocellular carcinoma (HCC). HBV genotypes are not only predictive of clinical outcomes but have also been associated with response to antiviral therapy [4], hepatitis B e antigen (HBeAg) sero-conversion rates and mutational patterns in the pre-core and core promoter regions [5].

HBV is classified into eight genotypes (A–H) based on a divergence in the entire nucleotide sequence >8 %. Furthermore, on the basis of a >4 % but <8 % divergence in the complete nucleotide sequence, each genotype can be sub divided to sub genotypes [6]. The HBV genotypes are proving to be an invaluable tool in tracing the molecular evolution and patterns of spread of HBV. They show a distinct geographical distribution between and even within regions. Structural and functional differences between genotypes can influence the severity, likelihood of complications, HBeAg sero-conversion and response to vaccine and or treatment of HBV infection [7−9].

Concise studies have been carried out on the varying prevalence of HBV genotypes in North America, Asia, and Europe and even in some parts of Africa. Five HBV genotypes (A–E) have been identified in Africa with some particular genotypes predominantly found in some countries like genotype A in Kenya [10], genotype D in Tunisia [11], genotype A–D in South Africa [12] and genotype E in Nigeria [13]. However, Cameroon with an HBV prevalence rate >8 % [14] still does not have enough information on HBV genotypes prevalent across the country. This study seeks to determine the prevalence of HBV genotypes in the North West and South West regions of Cameroon and further investigate the influence of HBV genotypes on viral loads as has been shown in studies elsewhere [15].

An increase in HBV viral load may determine the degree of liver damage and subsequent development of HCC [16]. Alanine aminotransferase (ALT) and aspartate aminotransferase (AST) which are usually released into the blood circulation following liver cell damage are also used as markers for disease progression in Hepatitis [17]. This study also investigates the relationship between HBV viral load and serum aminotransferase levels. This comparison can be used to justify the validity of ALT/AST measurement as a single marker for HBV disease progression as is commonly used in developing countries like Cameroon. In addition, because ALT and AST released during HBV infection may be dependent on HBV genotype, this study compares the effects of the different HBV genotypes identified on ALT and AST levels.

## Methods

### Study design, site and population

This was a hospital-community based cross-sectional study which enrolled participants who were 18 years of age and older. The Participants were enrolled from the Bamenda Regional hospital, Bamessing rural community and St. Theresa Medical Centre, Mambu-Bafut of the North West region while in the South West region participants were enrolled from Buea Regional Hospital, Limbe Regional Hospital, Muyuka health district, Muea heath centre and Tiko. Some of the HBV positive participants had been previously diagnosed of HBV (using Diaspot strips which detect HBsAg) during a free screening community exercise that took place 8 months before this study. The other participants knew their status at least 6 months before this study was conducted. These chronic HBV positive patients were screened again for HCV, HIV as well as HBV. Patients who showed HBV–HIV or HBV–HCV co-infection were excluded from the study. Fifty volunteers who were serologically negative for HIV, HBV and HCV were also enrolled from the South West region and used as “healthy controls” in the study. Prior to testing, the participants went through pre-test counselling by competent HIV/AIDS counsellors.

### Ethical considerations

The Cameroon National Ethics Committee (CNEC) in Yaoundé as well as the authorities in the concerned hospitals approved this study. Each participant signed a consent form at enrolment. For participants who were unable to read or understand the consent, a trained and competent counsellor verbally explained the information.

### Sample collection and analysis

Approximately 10 ml of venous blood was collected (by a trained phlebotomist) in 2 separate 5 ml tubes (one containing EDTA and the other with no anticoagulant) from each participant using vacutainers. Samples without EDTA were centrifuged at 1000*g* for 5 min and sera aliquoted. The sera was used for HBV, HCV and HIV screening using immuno-chromatographic tests (Diaspot, Acon^®^ Laboratories Inc and Abbot Determine for HBsAg, HCV antibody and HIV antibody detection respectively) following manufacturer’s instructions. as well as for determining levels of AST and ALT [AST-(GOT)-HBE06 and ALT-(GPT)-HBE07, Cypress Diagnostics Ltd.] by spectrophotometry (Mindray^®^ BA-88 Biochemistry analyser) following the manufacturer’s instructions. The liver enzyme tests were carried out on both hepatitis B positive and healthy control cases. The EDTA samples were centrifuged, plasma aliquoted and shipped in dry ice to BioCollections Worldwide Inc; Miami, Florida, USA for HBV viral load and genotyping. Shipped plasma specimens were further screened as well for HBV, HCV and HIV using Genetic systems hepatitis B surface antigen enzyme immuno-assay (Bio-Rad Laboratories, Washington DC, USA), Chiron RIBA HCV 3.0 strip immuno-blot assay (Ortho Diagnostic Systems, New Jersey, USA) and GS Western blot (Bio-Rad Clinical diagnostics) respectively according to manufacturer’s instructions. This was done to confirm the previously acquired results obtained via the immunochromatographic techniques. Viral load and genotyping was performed only for HBV-mono infected cases using the Abbott Real*Time* HBV automated *m*2000 system and INNO-LiPA HBV Genotyping assay respectively. We took into consideration the identified shortcomings of using INNO-LiPA HBV Genotyping assay which could lead to overestimation of mixed HBV genotypes [18].

### Statistical analysis

Data was input into Microsoft Office Excel 2013 and analysed using SPSS for Windows version 17. Means of enzyme levels were compared with independent sample *t* test and the Chi-square test was used to compare categorical variables. Two sided p value < 0.05 was considered significant for all analysis.

## Results

Demographic characteristic of patients and controls recruited in the study are shown on Table 1.

**Table 1:** Demographic characteristics of study participants (N = 131)

Characteristic	Categories	HBV positive cases, N = 81, n (%)	Healthy control, N = 50, n (%)
Region	North West	31 (38.3)	–
	South West	50 (61.7)	50 (100)
Sex	Male	29 (35.8) Mean age: 30.4 ± 9.8 SD Median age: 27 years	30 (60) Mean age: 41.5 ± 5.1 SD Median age: 41 years
	Female	52 (64.2) Mean age: 31.9 ± 7.7 SD Median age: 30 years	20 (40) Mean age: 36.9 ± 9.8 SD Median age: 39 years
Age group (years)	<30	42 (51.9)	06 (12)
	≥30	39 (48.1)	44 (88)

*SD* standard deviation

### Comparison of AST and ALT levels in HBV cases and healthy controls

The mean alanine aminotransferase (ALT) and aspartate aminotransferase (AST) levels were significantly higher (p < 0.001) in HBV infected patients than healthy controls. However, these values (AST: 16.69 ± 6.88 SD and ALT: 15.39 ± 7.08 SD) were significantly lower than upper limits of the test kits (AST ≥ 19 IU/ml, ALT ≥ 22 IU/ml). After adjusting for age and sex, AST (p = 0.001) and ALT (p < 0.001) were still higher in HBV infected cases than controls (Table 2).

**Table 2:** Comparison of mean ALT and AST levels between HBV infected cases and controls

Enzyme (IU/mL)	Cases N = 81 Mean ± SD	Control N = 50 Mean ± SD	Mean difference [SE]	p value	Adjusted mean difference [SE]^a^	p value
AST	16.69 ± 6.88	12.78 ± 4.60	3.91 [1.10]	0.001	4.21 [1.19]	0.001
ALT	15.39 ± 7.08	9.94 ± 4.17	5.45 [1.10]	< 0.001	5.69 [1.31]	<0.001

*SD* standard deviation, *SE* standard error, *AST* aspartate aminotransferase, *ALT* alanine aminotransferase

^a^Adjusted for age and sex

Generally, abnormal AST levels (M > 19, F > 16 IU/ml) were common in HBV infected cases than controls (35.8 vs 10 %, p = 0.001). Similarly, abnormal (M > 22, F > 18 IU/ml) ALT level was higher in infected cases than controls (17.3 vs 2 %, p = 0.008).

### Viral load and genotype distribution

Of the 81 HBV infected cases viral load was detected in 76 (93.8 %) with mean viral load of 120,807 IU/ml with a standard deviation (SD) of ± 440,159 IU/ml (range 14–2,664,237) while 5 (6.2 %) of the participants had undetectable viral load. Eleven (14.5 %) of the patients had viral load ≥20,000 IU/ml (mean 826,715 ± 901,130 SD), while 65 (85.5 %) had viral load <20,000 IU/ml (mean 1345 ± 2775 SD).

Genotyping was performed on HBV patients with detectable viral load. The identified genotypes in order of prevalence were A (47.4 %), E (39.5 %), C/E (3.9 %) A/C (2.6 %), A/E (2.6 %), B (1.3 %), A/B (1.3 %) and B/C (1.3 %). Genotypes A and E were more prevalent (p = 0.034) in South West and North West region respectively. Age and gender distribution across the predominant genotypes A and E are stated on Table 3. Amongst all the mixed genotype infections, only A/E was found in the North West Region of Cameroon (Table 4).

**Table 3:** Gender and age distribution among genotype A and E infected persons

	Predominant genotypes (%)^a^		p value
	A	E	
*Age group*			
<30 years	21 (60 %)	14 (40 %)	0.243
≥30 years	15 (48.4 %)	16 (51.6 %)	
*Gender*			
Male	11 (44 %)	14 (56 %)	0.138
Female	25 (61 %)	16 (39 %)	

^a^Percentage within age group and within sex

**Table 4:** Distribution of HBV genotype cases based on patient address

HBV genotypes	South West region					North West region		
	Tiko	Buea	Limbe	Muyuka	Muea	Bafut	Bamenda	Bamessing
E	03	02	03	05	01	10	03	03
A	03	08	09	06	00	07	03	00
B	00	00	00	00	00	00	00	01
A/C	01	01	00	00	00	00	00	00
C/E	02	01	00	00	00	00	00	00
A/E	00	00	00	00	00	01	01	00
A/B	00	01	00	00	00	00	00	00
A/C	00	00	01	00	00	00	00	00

There was a significant difference in mean viral load of patients among the different genotypes (p = 0.031) with genotype E having the highest mean viral load. Male patients as well recorded a significantly higher mean viral load vis-à-vis female patients (p = 0.022) (Table 5). Similarly, patients with elevated AST (p = 0.002) and ALT (p < 0.001) levels had significantly higher viral loads than those with normal enzyme levels.

**Table 5:** Comparison of mean viral load in the age groups, sex, HBV genotypes, ALT and AST levels

Variables	Category	N	Mean ± SD (IU/ml)	p value
Age (years)	<30	39	107,555 ± 426,321	0.790
	≥30	37	134,775 ± 459,749	
Sex	Male	27	275,634 ± 701,475	*0.022*
	Female	49	35,493 ± 123,509	
Genotypes	A	36	26,855 ± 121,819	*0.031*
	E	30	293,221 ± 685,464	
	Others^a^	10	361 ± 340	
AST Levels	Normal	47	2259 ± 8036	*0.002*
	Abnormal	29	312,936 ± 676,014	
ALT Levels	Normal	62	9743 ± 41,075	*<0.001*
	Abnormal	14	612,659 ± 889,047	

p values that show a statistically significant difference are in italics

SD standard deviation, *AST* aspartate aminotransferase, *ALT* alanine aminotransferase, *AST* (abnormal levels: M > 19, F > 16 IU/ml), *ALT* (abnormal levels: M > 22, F > 18 IU/ml)

^a^Others (B, B/C, A/B, A/E, C/E, A/C)

Both mean AST and ALT levels were significantly higher in patients with viral load ≥20,000 IU/ml when compared to those with viral load levels <20,000 IU/ml. Likewise, AST levels were significantly higher in patients infected with genotype E when compared to A and others (Table 6). Viral load correlated significantly with ALT (σ = 0.425, p < 0.01) and AST (σ = 0.272, p = 0.018).

**Table 6:** Comparison of mean ALT and AST levels among the genotypes and viral load categories

Genotypes	Enzymes (mean ± SD, IU/ml)			
	AST	p value	ALT	p value
A	16.36 ± 5.69	*0.019*	15.08 ± 5.91	0.176
E	19.74 ± 8.37		17.49 ± 9.10	
others	13.69 ± 2.27		13.18 ± 4.56	
*Viral load category (IU/ml)*				
<20,000	15.97 ± 5.98	<0.001	14.29 ± 5.61	<0.001
≥20,000	24.34 ± 6.96		23.82 ± 10.01	

p value that shows a statistically significant difference are in italics

*SD*- standard deviation, AST-aspartate aminotransferase, *ALT* alanine aminotransferase

^a^Others (B, B/C, A/B, A/E, C/E, A/C)

## Discussions

### Genetic diversity of HBV and possible impact on disease progression, virulence and clinical outcome

HBV is a remarkable example of a virus that draws attention with its different genotypes (A–H) showing marked geographical distribution across the world. The investigation of genetic diversity of the viruses in different locations is important, because variants may differ in their patterns of serologic reactivity, pathogenicity, virulence, and response to therapy [19]. Many studies conducted in the past have revealed genotype E predominance in West Africa. In this study, the most prevalent genotypes were A (47.4 %) and E (39.5 %). Genotype A (55.3 %) was more prevalent in South West while genotype E (55.2 %) was more prevalent in the North West region of Cameroon. Our finding alongside other similar findings of research conducted in different parts of Cameroon [20, 21, 22] may suggest that these genotypes (A and E) originated from this part of the world and are rapidly being transmitted here. In this study, Genotype E was even found in all the major areas were the patients resided (Table 4) indicating a wide distribution of the genotype in the North West and South West regions of Cameroon. The high prevalence of genotype A and E shows limited heterogeneity of HBV genotypes in Cameroon and this may imply limited variations in the progression, clinical outcomes and antiviral therapy of HBV infections in Cameroon. As seen in other studies [23], mixed genotype infections were as well recorded in this study with 77.8 % (7/9) of such coming from the South West region. Genotype A/C and C/E were identified in patients from the same town or neighbouring towns (Buea and Tiko) in the South West Region (Table 4) suggesting that these may be cases coming from a common source and circulating at low levels within this area. Likewise, mixed genotype A/E found only in Bamenda and neighbouring Bafut in the North West Region of Cameroon may as well imply that this mixed genotype originated from the same individual residing in that area.

The HBV DNA level, which is directly proportional to the amount of HBV in the patient, is considered the gold standard for determining disease progression and has recently been shown to be the most useful predictor of the development of cirrhosis and hepatocellular carcinoma (HCC) [24]. In our study, HBV genotype E was significantly associated with high mean viraemia and mean AST. This observation makes it increasingly obvious that heterogeneity in the global distribution of HBV genotypes may be responsible for differences in the progression, clinical outcomes of HBV infections and the possible response to vaccines and antiviral treatment as seen in some other studies [15]. However, genotype E which happens to be predominantly found in West Africa, has not been extensively studied in terms of its pathogenicity and virulence [20, 25].

### HBV viral load and liver aminotransferase

The release of liver aminotransferase into blood circulation following liver cell damage or lysis of hepatocytes is believed to be mostly associated with cell mediated immunity rather than the cytopathic effect of the virus [26]. This liver cell death may be either via apoptosis or via necrosis. Apoptosis in infected hepatocytes (orchestrated by T-cytotoxic cells via the perforin/granzyme pathway) is characterized by little or no cell content spillage into the circulation (following death) since the apoptotic bodies are usually phagocytosed and internally destroyed by macrophages unlike in necrosis where the cells experience unfavourable conditions, swell and burst releasing their content into the circulation. [27]. With this in mind, one may expect to find more AST and ALT in circulation following necrosis rather than apoptosis. However, there is still a challenge in determining which modes of cell death predominate in various forms of liver disease and injury. Apoptosis and necrosis frequently represent alternate outcomes of the same cellular pathways to cell death [28]. A statistically significant difference between the ALT and AST means of HBV infected cases and healthy controls justify aminotransferase elevation in circulation during HBV infection following liver damage as demonstrated by many other studies [29–31].

One may probably expect a patient with a high viral load to have a high number of HBV infected hepatocytes and as such more liver aminotransferase in circulation following the damage of these numerous infected cells. As observed in other studies [32], our study was very much in line with this theory as patients with abnormal (high) ALT and AST values showed significantly greater mean viral loads as compared to patients with normal ALT and AST values.

However, this identified relationship does not necessarily justify the use of liver aminotransferases as single biomarkers for liver damage and/or disease progression as is done in Cameroon and other resource-limited settings because some patients with normal ALT and AST also recorded high HBV viral loads. This implies that perhaps a good number of the cells died via apoptosis and as such, did not spill AST and ALT into the circulation. A liver tissue biopsy can as well reveal inflammation in such cases [27]. Looking at the different phases of chronic Hepatitis B infection, one could either group these particular patients under chronic immunotolerant phase (HBeAg positive, high HBV viral load and normal liver aminotransferase levels) or chronic immune active wild type HBV infection (HBsAg positive longer than 6 months, HBeAg positive, anti-HBe negative, high HBV viral load, elevated or normal liver aminotransferase levels). Both cases are characterized by the presence of HBeAg which is an indicator of active HBV replication and could imply high infectivity and transmission [33]. However, it becomes a bit challenging because some chronic HBV infections with active replication (elevated HBV DNA levels) are HBeAg negative (infection from precore mutant HBV strains) and this can be identified as active by the use of revised cut off values for ALT and HBV DNA levels as proven by some other studies [29–31]. Our study could not justify this because complete HBV serological profile test was not conducted on its participants to determine presence or absence of HBeAg and this is a key limitation to the study.

## Conclusion

HBV genotype A and E are most common in the South West and North West regions of Cameroon respectively. Genotype E was significantly associated with higher mean viral load and mean AST levels. However, this study suggest that liver aminotransferase levels and HBV viral load may not always show a direct proportional relationship as some patients had normal aminotransferase levels but high viral loads, which could be considered as active HBV infection. Therefore, viral load and liver aminotransferases measurement alongside HBV profile are recommended markers for HBV disease staging and progression.

## Abbreviations

ALT: alanine aminotransferase
AST: aspartate aminotransferase
CDC: Center For Disease Control and Prevention
DNA: deoxyribonucleic acid
EDTA: ethylenediaminetetraacetic acid
HBsAg: hepatitis b surface antigen
HBeAg: hepatitis B “e” antigen
HBV: hepatitis B virus
HCV: hepatitis C virus
HIV: human immunodeficiency virus
RIBA: recombinant immunoblot assay
CNEC: Cameroon National Ethics Committee
PCR: polymerase chain reaction
CI: confidence interval
SD: standard deviation
SPSS: statistical package for social sciences
HCC: hepatocellular carcinoma
WBC: white blood cell
